# Investigation of healthy living strategies in elderly who achieved to live long and healthy

**DOI:** 10.12669/pjms.36.3.1838

**Published:** 2020

**Authors:** Turgut Sahinoz, Saime Sahinoz

**Affiliations:** 1 Dr. Turgut Sahinoz, M.D., Public Health Specialist, Assist. Professor, Department of Health Management, Ordu University, Faculty of Health Sciences, Altinordu, Ordu, Turkey; 2 Prof. Dr. Saime Sahinoz, M.D., Public Health Specialist, Department of Emergency and Disaster Management, Gumushane University, Faculty of Health Sciences, Merkez, Gumushane, Turkey

**Keywords:** Long life, Healthy life, Elderly

## Abstract

**Objective:**

To determine the lifestyles, healthy living strategies and socio-cultural characteristics of elderly people who lived long and healthy.

**Methods:**

This study was a cross-sectional study. This study was conducted on 472 elderly patients, aged 80 years and over, selected by random sampling method. The socio-demographic characteristics and daily living activities of the elderly were determined by face to face interview technique using the “Elderly Questionnaire” developed by the researchers in 2018. Chi-square test was used for statistical analysis.

**Results:**

Based on the average age (84) of the elderly in the research group, it was seen that they were able to exceed the average life expectancy of their fathers (72) and mothers (73). It has been found that the participants live 11-12 years longer than their parents. It was also found that more than half (51.9%) of the elderly have the habit of walking regularly every day. It was determined that the elderly mostly consumed vegetables and fruits (88.5%), milk and dairy products and meat, respectively.

**Conclusion:**

The elderly stated that natural and healthy nutrition, working and staying away from stress in the top three places as the reason of their long and healthy life.

## INTRODUCTION

As life expectancy is increasing one of the most important issues in today’s is how to maintain healthy aging and the health of the elderly. Aging is an inevitable phenomenon in physiological terms. Generally, the limit is considered as 65 years. The transition to a dependent state usually occurs after the age of 75 years. 1, 2 The ratio of individuals aged 65 years and over consisted of 7.1%, 7% and 7.3% of the total population in 2007, 2009 and 2011, respectively in Turkey and it is expected that it will reach to 9% by 2025.^3^ Aging does not only have disadvantages, but if you take care of healthy aging, the old age can be lived very well.^2^

In order to stay healthy and feel good it is needed to maintain a healthy lifestyle (adequate and balanced nutrition, active lifestyle, establishing social relations, etc.), and to take the necessary measures in time.^4^ The World Health Organization reports that the unhealthy lifestyle (smoking and alcohol use, malnutrition, sedentary living, etc.) is responsible for one third of the burden of chronic illness.^1^ Protecting the health of the elderly is one of the most important goals of public health.

The fact that people live longer has brought along the desire to spend this period healthier. Longevity is associated with genetic material, but genes are not the entire story.^5^ Nutrition, life-style, social support all contribute to longevity.^6^

This research has been planned on the thesis that to determine what kind of lifestyle should be adopted for healthy aging, the life styles of people who have managed to live healthily over the age of 80 should be taken into consideration.In this study; it has been aimed to determine the lifestyles and socio-cultural characteristics of elderly people who lived long and healthy and to establish a lifestyle for healthy aging which will serve as an example for future generations.

## METHODS

This study is a cross-sectional study. Life expectancy at birth in Turkey was 78 years for 2017.^3^ The study was planned for the elderly living longer than the average life expectancy at birth, so elderly people aged 80 and over were included in the research group. Again, the research was planned on healthy elderly people. The elderly were evaluated as healthy if they did not have any diagnosed diseases according to their own statements. Before starting the research, (30.10.2018 Number. 2018/8) ethical approval was obtained from the ethics committee of Gumushane University. The universe of the study consisted of healthy elderly people aged 80 and over living in the central district of Gumushane province in Turkey in 2018. Gumushane province is located in the North, in the Black Sea region of Turkey. The sample size was calculated from the online software raosoft.com by taking 95% CI and 5% of margin of error required for sample of 472. The sample consisted of 472 elderly individuals selected by simple random sampling method living in the central district of Gumushane province. The “Elderly Questionnaire” was developed by the researchers in accordance with the literature. The reliability of the questionnaire was 0.725 Cronbach’s alpha. The Kendall Coefficient of Concordance W correlation test was applied to determine the questionnaire’s validity and it was found to be valid (p> 0.05). This study was conducted on 472 elderly patients with good general health status, aged 80 years and over, selected by random sampling method, not bed-dependent, with sufficient cognitive functions and willing to participate in this study. The socio-demographic characteristics and daily living activities of the elderly were determined by face to face interview technique using the “Elderly Questionnaire” developed by the researchers in Gumushane province in November 2018-December 2018. The data were analyzed by statistical package program on computer. Chi-square test was used for statistical analysis. p≤0.05 was considered statistically significant.

## RESULTS

The mean age of the study group was 84.05±3.23 (min=80, max=108) years. Other characteristics of study subjects are given in Table-I.

**Table-I:** Some characteristics of the elderly in the research group.

Characteristic		Number	%
Gender	Male	294	62.2
	Female	178	37.8
Marital Status	Married	339	72.1
	Never Married	4	0.9
	Spouse (wife or husband) is dead	119	24.9
	Divorced	10	2.1
Educational Status	Illiterate	87	19.5
	Literate	22	4.7
	Primary School	259	54.6
	Secondary School	32	6.8
	High School	52	10.6
	University Associate Degree	4	0.7
	University Degree	16	3.1
Residential Place	Rural	155	32.8
	Urban	317	67.2
Housing Type	Detached House	284	60.0
	Apartment	188	40.0
Family Type	Nuclear	303	64.0
	Extended	163	34.9
	Shattered	6	1.1
Economic Status of the Family	Good	289	61.1
	Medium	158	33.5
	Bad	25	5.4
House	Present	410	86.8
	Absent	62	13.2
Auto	Present	102	21.6
	Absent	370	78.4
Help Request	Present	59	12.5
	Absent	413	87.5

Total		472	100.0

The mean age at the time of death of the fathers of the elderly in the research group was 72.05 ± 14.63, and the mean age of death of their mothers was 72.74 ± 15.33. 12.6% of the elderly were living alone, 43.7% were living with their spouse, 22.8% were living with their spouse and children, 19.4% were living with their children, 0.2% were living in elderly nursing home, 1.3% were living in different places.

About 82.4% of the elderly stated that they were satisfied with their lives. When they were asked as to how you evaluate your own health; 10.9% of the elderly answered very well, 36.6% good, 39.1% medium and 13.4% bad.

A statistically significant difference has been found between life satisfaction of the elderly according to their self-health assessment status (p=0.0001). It has been found out that when the self-health assessment status was good and very well elderly were more satisfied. The elderly respond to the question of “Can you get out of the house?” as 51.9% go for a walk every day. About 13.4% of the elderly are smokers. 1.9% of the elderly use alcohol. When asked about the opinions of ready-made food products, 60.1% of the elderly replied that ready-made foods are unhealthy and should not be consumed because they contain chemicals. It was determined that the elderly mostly consumed vegetables and fruits, milk and dairy products and meat, respectively. Almost 88.5% of elderly people consumed natural vegetables and fruits according to the season.

The elderly stated natural and healthy nutrition, working and staying away from stress in the top three places as the reason of their long and healthy life. About 84.5% of the elderly stated that they offered prayer five times a day.

## DISCUSSION

In our study, based on the average age (84) of the elderly in the research group, it was seen that they were able to exceed the average life expectancy of their fathers (72) and mothers (73). It also showed that the participants live 11-12 years longer than their parents. When the average life expectancy for Turkey is considered to be about 78, it has been determined that the participants have already managed to live 6 years more compared to the national average. This means that our elderly people overcome existing genetic and environmental problems with good practices in their lifestyles.

**Table-II:** Body Mass Index of the elderly in the research group.

Status	Body Mass Index	Number	%
Weak	below 18.5 kg/m2	6	1.2
Normal weight	between 18.5-24.9 kg/m2	162	34.4
Overweight	between 25-29.9 kg/m2	202	42.8
Obese	over 30 kg/m2	102	21.6

Total		472	100.0

When the socio-cultural characteristics of the elderly in the research group were examined; 72.1% were married, 54.6% were primary school graduates, 67.2% were living in urban areas, 60% were living in detached houses, 64% were living in nuclear families, 68.8% owned the property of their own homes, only 21.6% were found to have cars. In addition to this data, 61.1% of the elderly stated that their family had good economic status and 87.5% of the elderly stated that they did not need any help. There is a positive relation between the life expectancy and economic growth in Turkey.^7^

If we interpret the data; being married, living in detached houses, living in a nuclear family, owning their own house, not having a car are positive conditions for healthy aging. High level of education affects health in a positive way. Although the majority of the elderly in the research group are primary school graduates and this is in contradiction, considering the period in which they live, their level of education can be considered good. Another contrast is that the majority is living in urban areas. Living in urban areas has both negative and positive effects on healthy aging. It is generally known that those living in rural areas live longer and healthier. However, living in urban areas is an advantage in terms of access to health services. In a study life-long vaccination is recommended to promote healthy ageing.^8^

**Table-III:** Life satisfaction compared with self-health assessment status.

Self-Health Assessment Status	Life Satisfaction					

	Satisfied		Not satisfied		Total	

	Number	%	Number	%	Number	%
Good+Very Well	212	94.2	13	5.8	225	100.0
Medium	156	84.2	29	15.8	185	100.0
Bad	23	37.1	39	62.9	62	100.0

Total	391	82.7	81	17.3	472	100.0

It is interesting that the majority of the elderly state that their families have a good economic situation and that they do not need help. This finding shows us that being contented has a positive effect on long and healthy life. Again 84.5% of the elderly stated that they offered prayer five times a day. Again, 82.4% of the elderly stated that they were satisfied with their lives. Prayer is very beneficial for both physical and mental health. In many studies significant relationships between prayer and self-esteem, depression and suicide probability are reported and a positive correlation was found between religiosity and improved health status.^9^-^12^

A statistically significant relationship was found between the feeling of being healthy and life satisfaction of the elderly (p = 0.0001). It was also found that elderly people who say that their health status is good are more satisfied with their lives. Being satisfied with your life and giving thanks to God is a very important factor for a healthy life. When the lifestyle of the elderly in the research group is examined; It is known that the most important factor in terms of healthy lifestyle is natural, adequate and balanced nutrition which is the foundation of health.^13^ About 65.5% of the elderly pay attention to consume natural foods, 60.1% stated that ready-made foods are unhealthy and should not be consumed because they contain chemicals. Nowadays, it is known that many diseases develop due to additives in ready-made foods.^13^ These data indicate that long and healthy living depends on not consuming ready-made foods.

When the elderly were asked “What kind of foods do you consume most?” It was determined that they mostly consumed vegetables and fruits, milk and dairy products and meat, respectively. About 88.5% of elderly people consumed natural vegetables and fruits according to the season. In a study conducted at centenarians it was found out that yogurt consumption was frequent among the participants and this result is consistent with the results of our study.^14^

We also found that more than half (51.9%) of the elderly have the habit of walking regularly every day, which is an important element in daily living activities. Walking regularly every day reduces morbidity and mortality rates and leads to a long and healthy life. Adequate physical activity is a very important factor in keeping heart health, blood pressure, blood sugar and cholesterol within the normal limits.^15^ It is estimated that 31519 deaths due to ischemic heart disease and 10269 deaths due to ischemic stroke can be prevented if the physical activity habit is sufficient in our country.^16^

Only 21.6% of the elderly in the study group were found to be obese. This rate is lower than the general obesity prevalence in population. Moderate weight loss has been reported to have positive outcomes on functional capacity, independence and management of chronic diseases in the elderly. In a study conducted in our country, the frequency of obesity in the elderly was reported to be 48.7%.^17^ In another study it has been found out that the individuals who lost weight through adopting healthy dietary habits had increased Short Form (SF-36) Quality of Life Scale scores.^18^ The elderly stated that natural and healthy nutrition, working and staying away from stress in the top three places as the reasons of their long and healthy life.

In yet another study at centenarians it was found out that worship and prayer was frequent among the participants and the reason for reaching advanced age was stated as natural diet, avoiding bad habits and living in a peaceful environment.^14^ In some other studies it has been found out that natural nutrition is effective on healthy and long living.^19^-^22^ The results of a survey indicate that consumers have insufficient knowledge about lifestyle diseases, including osteoporosis and diet of large part of society is not properly balanced which can obstruct proper bone mineralization.^23^ In a study conducted to determine healthy lifestyle behavior and affecting risk factors in workers at small and medium-sized enterprises from four different sectors in Aydin, Turkey, it has been found out that the workers got low scores for physical activity, but high scores for spiritual development.^24^ Yet another study conducted for evaluation of nutritional status of elderly patients presenting to the Family Health Center showed that 38.2% of these patients were found to have malnutrition, 18.6% were at high risk of malnutrition and 43.1% had a normal nutritional status. The findings suggest evaluating nutritional status in individuals aged 65 years and older in the population on a regular basis to reduce disease risk and mortality.^25^

## CONCLUSION

This study showed that the participants have been able to live 11-12 years longer than their parents and six years longer than the national average. It was found that more than half (51.9%) of the elderly have the habit of walking regularly every day, 84.5% of the elderly stated that they offered prayer five times a day. Again, 82.4% of the elderly stated that they were satisfied with their lives. It was found that elderly people who say that their health status is good are more satisfied with their lives. 65.5% of them pay attention to consume natural foods, 60.1% stated that ready-made foods are unhealthy and should not be consumed because they contain chemicals.

The reasons of long and healthy life of the healthy elderly who live a life above the average life expectancy in Turkey is found to be natural and healthy nutrition, working and staying away from stress. According to these results; to ensure a longer and healthier life for the society; natural and healthy nutrition, working and staying away from stress is recommended.

Being married, living in detached houses, living in a nuclear family, owning your own house, not having a car are found to be positive factors for healthy aging. The findings of the research show that long and healthy living is associated with not consuming ready-made food. It is interesting to note that the majority of the elderly state that their families have a good economic situation and that they do not need help. This finding shows us that being content has a positive effect on long and healthy life.

### Authors Contribution:

**TS:** Conceived, designed and did statistical analysis, data collection, manuscript writing, editing of manuscript, review and final approval of manuscript, is responsible and accountable for the accuracy or integrity of the work.

**SS:** Did statistical analysis, data collection, manuscript writing, editing of manuscript, review and final approval of manuscript.
